# A prospective trial investigating the role of Serum 25-Hydroxyvitamin D in diagnosis and prognosis of bladder cancer

**DOI:** 10.1371/journal.pone.0266371

**Published:** 2022-06-16

**Authors:** Ahmed Abdelgawad, Abdelwahab Hashem, Ahmed Mosbah, Laila A. Eissa

**Affiliations:** 1 Biochemistry Department, Faculty of Pharmacy, Mansoura University, Mansoura, Egypt; 2 Urology Department, Urology and Nephrology Center, Mansoura University, Mansoura, Egypt; CIEMAT, SPAIN

## Abstract

**Purpose:**

Higher levels of serum 25-hydroxyvitamin D 25(OHD) are associated with better prognosis in breast and colorectal cancer. However, the evidence is still inconclusive for bladder cancer (BC). Herein, we investigated the diagnosis and prognosis roles of serum levels of 25(OHD) in suspected BC patients presented by hematuria.

**Methods:**

This prospective cohort study involved suspected patients of BC presented with hematuria. Patients were evaluated by CT urogram, office cystoscopy and urine cytology with subsequent inpatient biopsy for positive findings. Baseline blood samples were collected for measurement of 25(OHD) by electrochemiluminescence binding assay at the time of diagnosis. Patients with non-muscle-invasive BC (NMIBC) underwent transurethral resection of bladder tumor (TURBT) and adjuvant intravesical chemotherapy or BCG instillation. Patients were followed up for their recurrence status during 10 to 24 months. Recurrence was defined as the first time of NMIBC pathological relapse during the follow up period.

**Results:**

A total of 115 patients were included in the final analysis. Patients had proven pathological BC (64 with NMIBC, and 20 with muscle invasive) and 31 patients were considered as control group. Controls were those patients with BC-free workup (including cytology, cystoscopy, and upper tract imaging). BC group showed a lower level of 25(OHD) than control group 16.47±5.88 versus 28.99±3.19 ng/mL (p<0.001). In addition, muscle invasive group also showed a lower level than NMIBC group 13.17±4.5 versus 17.49±5.04 ng/mL (P = 0.003). During the follow-up period of, tumor recurrence occurred in 16 (25%) of NMIBC patients. The baseline 25(OHD) were decreased in patients who experienced early recurrence; without being statistically significant (15.99 ± 5.17 vs. 18.38 ± 5.14 ng/mL; p = 0.08). 25(OHD) deficiency/insufficiency occurred in 5 (16.1%) and 64 (76.2%) in control and BC patients, respectively, (odds-ratios (OR): 2.13; 95% confidence intervals (CI), 1.52–2.99; P < 0.0001).

**Conclusion:**

Serum 25(OHD) is significantly decreased in BC patients especially those with tumor muscle invasive group. However, the baseline serum 25(OHD) does not predict the recurrence in the NMIBC patients.

## Introduction

Bladder cancer (BC) accounts for 3% of global cancer diagnoses and the incidence rates are highest in Europe, the United States and Egypt. Approximately 75% of patients with BC are detected in early stages with disease confined to the mucosa or submucosa as non-muscle-invasive BC (NMIBC) [1]. Transurethral resection of bladder tumor either by conventional or enbloc technique is an important initial diagnostic and therapeutic tool in the management of NMIBC and risk-based intravesical therapy [2].

The oncological benefit of adjuvant intravesical therapy is particularly useful in patients with risk of recurrence to decrease this risk [3]. Low- and intermediate-risk NMIBC had a 5-year recurrence free survival rates of 43% and 33%, respectively. High-risk NMIBC had a progress to muscle invasive disease in 21% of patients [4, 5].

Bacille Calmette-Guérin (BCG) is the preferred treatment for high-risk NMIBC. BCG is also an option for intermediate-risk NMIBC. BCG adheres and internalize into resident immune, normal, and tumor urothelial cells. Then BCG induces antigen presenting, cell-mediated induction of innate and adaptive immune responses [6]. Toll-like receptor (TLR) signaling is an important component of the BCG anti-tumor effect [7].

It is considered that activation of TLR by BCG promotes vitamin D signaling via increases in 1,25(OH)_2_D synthesis and increased expression of the vitamin D receptor. Activation of TLRs promotes a response that allows the immune system to recognize and respond to abnormal cells, so it could prevent the NMIBC recurrence and progression [8].

Higher levels of serum 25-hydroxyvitamin D (25(OHD)), >30 ng/mL, are associated with better prognosis in breast and colorectal cancer [9]. However, the evidence is still inconclusive for BC. Herein, we investigated serum levels of 25(OHD) in suspected BC patients presented by hematuria and their diagnostic and prognostic value in BC patients.

## Patients and methods

This is a single center prospective cohort study, had been conducted at Mansoura Urology and Nephrology Center, Egypt. Between August 2018 and May 2019, 140 Patients with hematuria were assessed for eligibility. Inclusion criteria included suspected patients of BC with gross or microscopic hematuria. Exclusion criteria were hematuria of other causes as trauma, stones, and infection, medical and nephrologic causes of hematuria, and patient having previous or current malignancy.

Preoperative evaluations included detailed medical history, physical examination, routine blood examination, urinalysis, renal and liver function tests, computed tomography with contrast scans. Outpatient office flexible cystoscopy and cytology were performed for patients with hematuria. BC-free patients by cystoscopy and cytology were considered as control patients.

Patients with positive/suspicious bladder lesion were inpatients admitted and underwent standard rigid cystoscopy and transuretheral resection of bladder lesions were done to confirm or exclude malignancy. Immediate postoperative instillation of chemotherapy was done using Epirubicin 50 mg (2mg/ml) within the first 24hours.

Histo-pathology assessment was revised by a single expert uro-pathologist following the American pathologist protocol version 2012 [10]. cytology was evaluated according to the Paris classification system [11]. Suspicious and malignant samples were considered positive results, while, hyperplastic and negative for malignancy samples were considered negative results. Patients were followed according to the European guidelines 2015.v.1 for receiving intravesical instillation therapy [12].

All procedures in this study involving human participants were in accordance with the 1964 Helsinki Declaration and its later amendments. Informed consent was obtained from all individual participants in this study after approval of the Institutional Review Board of faculty of Pharmacy ethical committee, Mansoura University (ID. R/2017-15).

### Sample preparation and serum 25 (OHD) measurement

Vitamin D was determined by electrochemiluminescence binding assay on Elecsys and cobas e 601 immunoassay analyzers (Roche Diagnostics GmbH, Sandhofer Strasse 116, D-68305 Mannheim).

The inter-series CV was of ≤ 20% and the sensitivity of the method was of (3–100) ng/ml. Vitamin D deficiency, insufficiency and sufficiency are defined as plasma 25 (OHD) concentration <12, 12–20, and >20 ng/mL, respectively, according to The Institute of Medicine guidelines [13].

#### Outcome measures

The primary endpoint was serum 25(OHD) levels and its diagnostic role in the staging and grading of BC patients through calculating the sensitivity, specificity, positive predictive value, and negative predictive value of serum 25(OHD). BC diagnosis was defined as discrimination of BC from BC-free (controls). BC stage was defined by differentiation of NMIBC from MIBC. BC grade was defined as discrimination of NMIBC grade into low and high grade according to WHO/ISUP 2004 grading system.

The secondary endpoint was the prognostic role of baseline serum 25(OHD) levels in the prediction of recurrence in patients with NMIBC at short-term follow-up. Recurrence was defined as the first time of tumor relapse regardless of tumor stage and grade.

### Statistical analysis

Statistical analysis was performed with the use of the IBM SPSS Statistics version 22.0 for Windows (IBM Corp., Armonk, NY, USA). Statistical analyses were performed using chi-squared tests, Student’s t-test, Mann–Whitney-U test, as appropriate. A p-value <0.05 considered to be statistically significant.

Receiver operating characteristic (ROC) analyses were performed to determine the best sensitivity, specificity, diagnostic accuracy, and the area under the curve (AUC) with its 95% confidence interval (95% CI) for differentiating between the different subgroups. Kaplan-Meier analysis was utilized to determine the recurrence-free proportion, with significance determined by log-rank test. Univariable and multivariable Cox regression analyses were used to evaluate the factors affecting recurrence free rate.

## Results

From August 2018 and May 2019, 140 patients met the eligibility criteria. After exclusion of patients with insufficient urine volume, invalid results, or error in processing of the samples, 115 patients were included in the analysis. Control patients were patients with a normal flexible cystoscopy, imaging, and cancer free cytology such as benign prostatic hyperplasia, chronic non-specific cystitis, polypoid cystitis, Bilharizial cystitis, and/or overactive bladder.

In this trial, 31 (27%) of included patients were bladder tumor free (controls) and the remaining 84 (73%) of patients had a pathological proven BC; 64 (55.7%) with NMIBC, and 20 (17.4%) with MIBC. Baseline patients and tumor characteristics demographics are shown in Table 1.

**Table 1:** Baseline patients and tumors characteristics.

**Patients’ baseline and tumor characteristics (N = 115)**	
**Age, years, mean (SD)**	62 (10)
**Gender ratio, male/female**	90 / 25
**Body mass index, Kg/m2, mean (SD)**	25.25 (5.5)
**Serum 25-hydroxyvitamin D (25-OHD), ng/dl, mean (SD)**	19.62 (7.23)
**Serum 25-hydroxyvitamin D (25-OHD), n (%)**	
Deficiency, <12 ng/mL	28 (20)
Insufficiency, 12–20 ng/mL	48 (34.3)
Sufficiency, >20 ng/mL	64 (45.7)
**Hematuria type, *n* (%)**	
Macroscopic	97 (84.3)
Microscopic	18 (15.7)
Asymptomatic	12 (10.4)
Symptomatic	6 (5.3)
**Smoking history, n (%)**	
Never	19 (16.5)
Former	58 (50.4)
Current	38 (33)
**Cystoscopy findings, *n* (%)**	
Free	31 (26.9)
Bladder mass	84 (73)
**Bladder cancer, n (%)**	
Non-muscle invasive	64 (76.2)
Muscle invasive	20 (23.8)
**Non-muscle invasive bladder tumor patients’ baseline and tumor characteristics (N = 64)**	
**Gender, *n* (%)**	
Male	50 (78.1)
Female	14 (21.9)
**Age, *n* (%)**	
< 60 years	28 (43.8)
60–70 years	33 (51.6)
> 70 years	3 (4.7)
**Body mass index, Kg/m2, *n* (%)**	
< 30	55 (85.9)
≥ 30	9 (14.1)
**Tumor number, *n* (%)**	
Single	22 (34.4)
Multiple	42 (65.6)
**Tumor site, *n* (%)**	
Posterior	13 (20.3)
Anterior	6 (9.4)
Lateral	8 (12.5)
Domal	5 (7.8)
Multiple sites	32 (50)
**Tumor size, *n* (%)**	
< 3 cm	16 (25)
≥ 3 cm	48 (75)
**Tumor stage, *n* (%)**	
Ta	0
T1	64 (100)
Associated CIS	0
**Tumor WHO 1973 grading system, *n* (%)**	
G2	31 (48.4)
G3	33 (51.6)
**Tumor WHO/ISUP 2004 grading system, *n* (%)**	
Low grade	21 (32.8)
High grade	43 (67.2)

Serum 25(OHD) deficiency/insufficiency occurred in 5 (16.1%) and 64 (76.2%) in control and BC patients, respectively, (odds-ratios (OR): 2.13; 95% confidence intervals (CI), 1.52–2.99; P < 0.0001). Serum 25(OHD) deficiency/insufficiency occurred in 48(75%) and 16(80%) in non-muscle and muscle invasive BC patients, respectively, (OR: 1.25; 95% CI, 0.47–3.31; P = 0.65). Serum 25(OHD) deficiency/insufficiency occurred in 11(52.4%) and 37(86%) in low- and high-grade NMIBC patients, respectively, (OR: 2.06; 95% CI, 1.07–3.94; P = 0.003).

**Fig 1:**
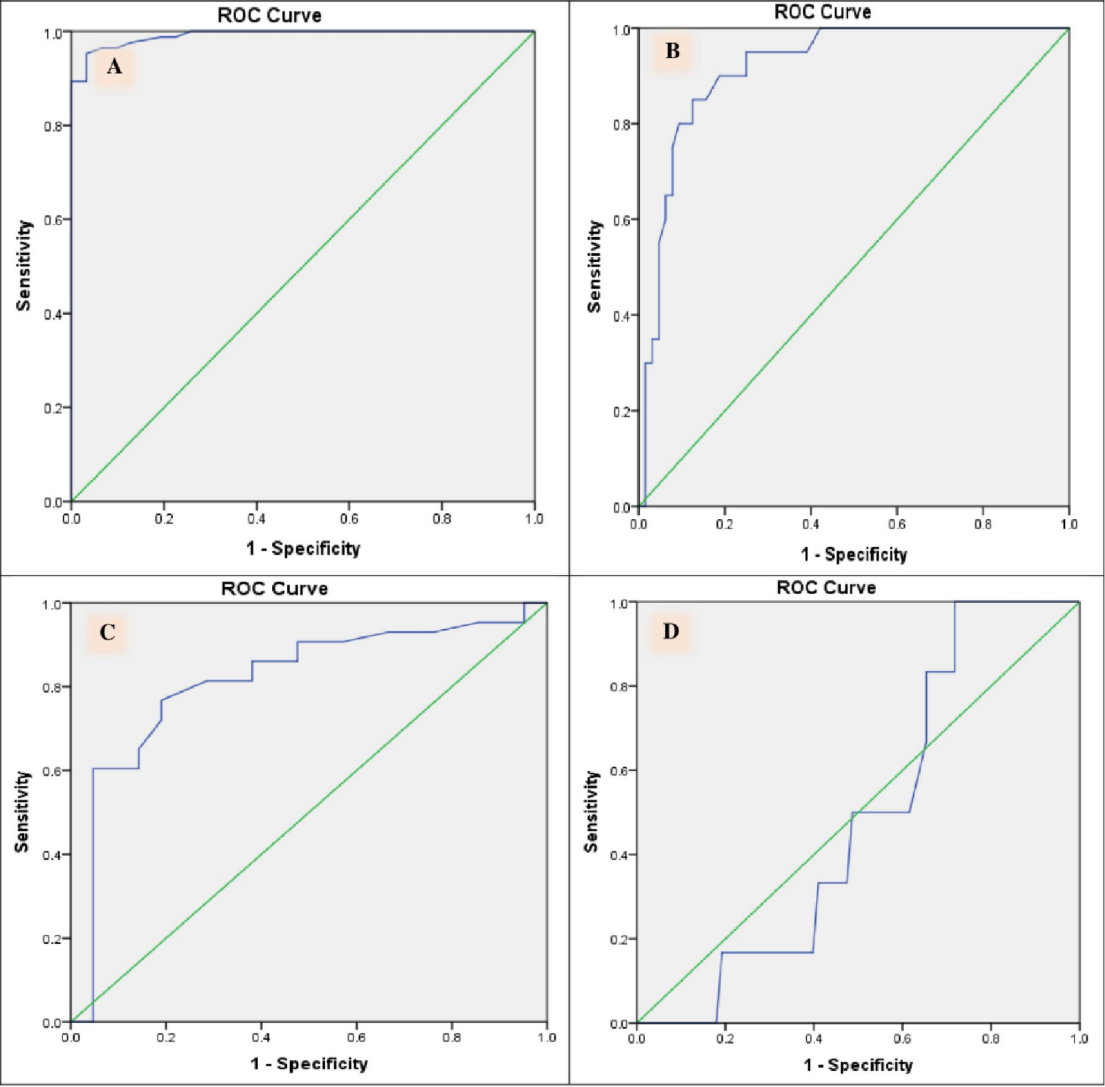
ROC curve for 25(OH)D in (A) bladder cancer patients vs. controls, (B) non-muscle invasive bladder cancer vs. Muscle invasive bladder cancer, (C) low-grade cancer vs. high-grade bladder cancer and (D) recurring bladder cancer vs non-recurrent bladder cancer.

By assessing the role of 25(OHD) in BC diagnosis, BC group had a lower level of 25(OHD) than control group 16.47±5.88 versus 28.99±3.19 ng/mL (p<0.001) (S1A Fig). Based on ROC analysis (Fig 1A), a cut-off value of 24.17 ng/mL discriminates BC patients from controls with sensitivity and specificity of 0.99 and 0.81, respectively (Table 2).

**Table 2:** Clinical performance characteristics of serum 25-hydroxyvitamin D in patients with hematuria for diagnosis, staging, grading of bladder cancer and prognosis of non-muscle invasive bladder cancer.

Test	AUC	Cut off	95% CI	Sensitivity	Specificity	PPV	NPV	Accuracy	P value
		ng/mL							
**Bladder cancer versus control (bladder cancer-free) group**									
25-OHD	0.991	24.17	98–100	**0.99**	**0.81**	**0.93**	**0.96**	**0.93**	**<0.001**
**Non-muscle invasive bladder cancer versus Muscle invasive bladder cancer**									
25-OHD	0.919	13.45	86–98	**0.95**	**0.75**	**0.54**	**0.98**	**0.58**	**< 0.001**
**High grade versus low grade non-muscle invasive bladder cancer**									
25-OHD	0.816	17.85	70–93	**0.77**	**0.81**	**0.89**	**0.63**	**0.78**	**< 0.001**
**Recurrent versus non recurrent occurrence during short-term follow up non-muscle invasive bladder cancer**									
25-OHD	0.487	15.3	32–66	**0.81**	**0.83**	**0.62**	**0.93**	**0.83**	0.917

AUC: area under the curve, CI: confidence interval, PPV: positive predictive value, NPV: negative predictive value.

MIBC patients showed a lower level than NMIBC patients’ group 13.17±4.5 versus 17.49±5.04 ng/mL, respectively (p<0.001) (S1B Fig). Based on ROC analysis (Fig 1B), a cut-off value of 13.45 ng/mL discriminates NMIBC from MIBC with sensitivity and specificity of 0.95 and 0.75, respectively (Table 2).

Regarding the serum 25(OHD) assumed task in discriminating NMIBC according to WHO/ISUP 2004 grading system into low and high grade, serum 25(OHD) showed a sensitivity (77%) and specificity (81%) for discrimination between low and high grade at a cut-off point 17.85 ng/ml (Fig 1C). However, serum 25(OHD) showed no significant difference between low and high grades patients (P = 0.25) (S1C Fig).

The median (interquartile range) of follow up period was 14 (11, 18) months for the NMIBC to detect the recurrence occurrence. Tumor recurrence occurred in 16 (25%) of those patients. Moreover, 25(OHD) showed no significant difference between recurrent and non- recurrent patients (P = 0.08) (S1D Fig). In addition, serum 25(OHD) did not predict the recurrence in NMIBC patients (Fig 1D).

**Fig 2:**
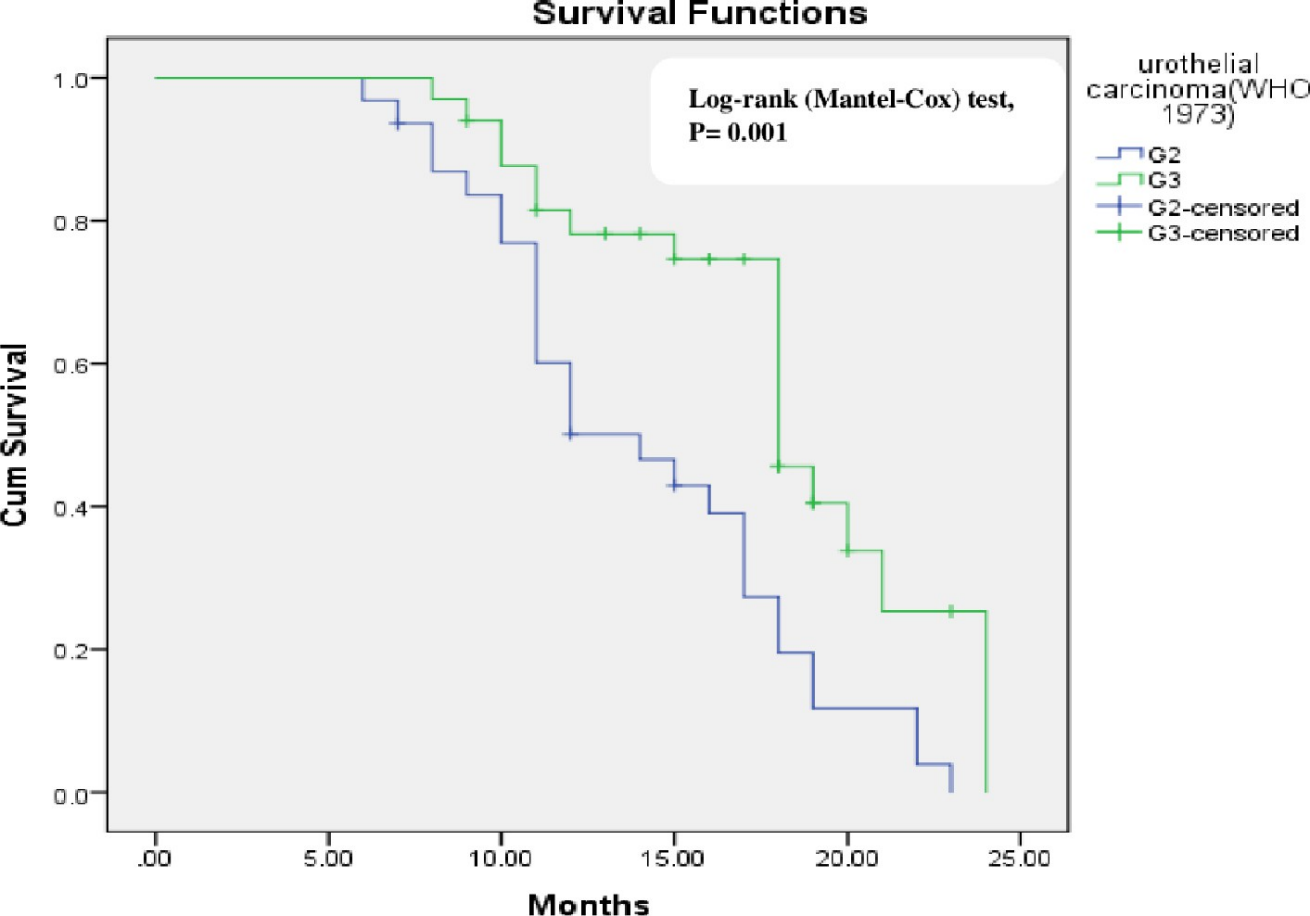
Kaplan-Meier survival curves showing recurrence-free survival of non-muscle invasive bladder cancer group in relation tumor grade (WHO/ISUP 2004 system).

**Fig 3:**
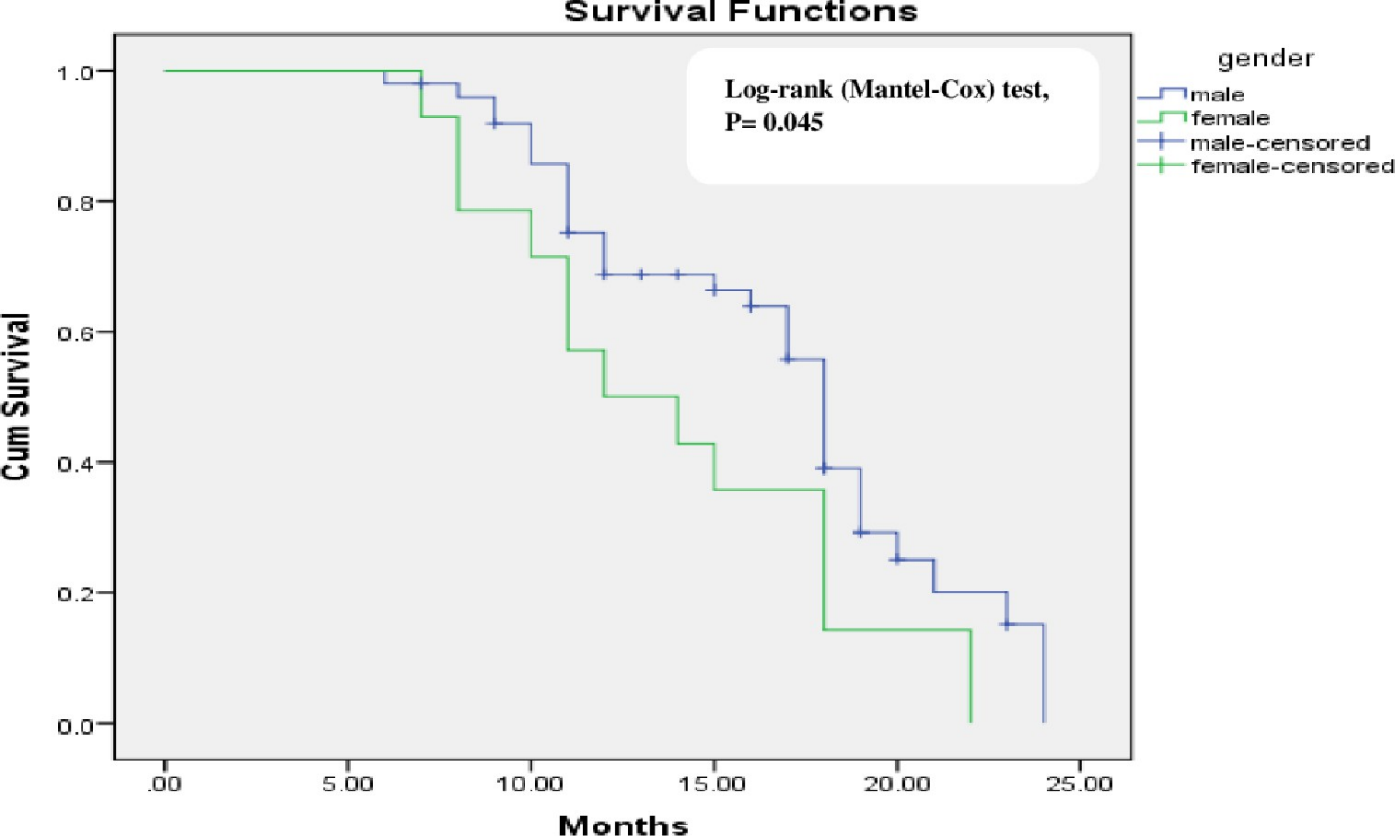
Kaplan-Meier survival curves showing recurrence-free survival of non-muscle invasive bladder cancer group in relation to gender.

Gender (P = 0.015) and tumor grade (WHO 1973 grading system) (P = 0.006) were the significant factor affecting tumor recurrence on univariate analysis (Table 3). Kaplan-Meier analysis that determine the recurrence-free proportion are shown in Figs 2 and 3. Using multivariable cox-regression analyses, gender did not significantly affect recurrence free survival [Hazard ratio (HR) (95% CI) = 0.69 (0.36–1.33) P = 0.27] and tumor grade was the only predictor for recurrence free survival [Hazard ratio (HR) (95% CI) = 0.44 (0.24–0,8) p = 0.008].

**Table 3:** Analyses for predictors of recurrence among NMIBC patients.

Variable	No recurrence (N = 48)	Recurrence (N = 16)	P- value
**Age, *n* (%)**			0.94
< 60 years	21 (43.8)	7 (43.8)	
60–70 years	25 (52)	8 (50)	
> 70 years	2 (4.2)	1 (6.2)	
**Gender, *n* (%)**			**0.015**
Male	34 (70.8)	16 (100)	
Female	14 (29.2)	0	
**Body mass index, Kg/m2, *n* (%)**			0.30
< 30	40 (83.3)	15 (93.8)	
≥ 30	8 (16.7)	1 (6.2)	
**Tumor size, *n* (%)**			
< 3 cm	14 (29.2)	2 (12.5)	0.18
≥ 3 cm	34 (70.8)	14 (87.5)	
**Tumor WHO 1973 grading system, *n* (%)**			**0.006**
G2	28 (58.3)	3 (18.8)	
G3	20 (41.7)	13 (81.3)	
**Tumor WHO/ISUP 2004 grading system, *n* (%)**			0.17
Low grade	18 (37.5)	3 (18.8)	
High grade	30 (62.5)	13 (81.3)	

## Discussion

In a pooled analysis of randomized trial and prospective cohort study, based on the inverse association between 25(OHD) and cancer risk, found that serum 25(OHD) concentrations ≥40 ng/ml was associated with > 65% reduction in risk of invasive cancers [14].

Sufficient vitamin D3 levels might play a role in the prevention or progression of BC. Zhao et al., [15] stated that serum 25(OHD) concentration is inversely correlated with BC risk. Vitamin D and its analogues are potential anti-proliferative agents, which decrease the high mitotic rate of BC, suppress tumor progression by stimulating apoptosis, reducing cell proliferation, and tumor invasion [15]. On the other hand, no statistically significant relationship has been found between serum vitamin D3 and BC in a Turkish study involving 101 BC patients and 109 controls [16].

In this present study, we observed significant lower serum levels of 25(OHD) in BC patients compared to BC-free (control) patients. Serum 25(OHD) deficiency/insufficiency occurred in 16.1% and 76.2% of control and BC patients, respectively. Serum 25(OHD) sufficiency was associated with lower risk for BC.

There is a paucity in the literature addressing serum 25(OHD) deficiency/insufficiency among NMIBC patients. NMIBC is highly immune-responsive disease with intravesical BCG as the standard treatment for intermediate and high-risk disease to decrease recurrence and progression. Intravesical BCG induces a cell-mediated immunity, secretion of inflammatory cytokines and attraction of lymphocytes [8]. Vitamin D may play an immuno-regulatory role following BCG vaccination [17].

Amaral et al. [18] found that low plasma 25(OHD) concentrations were associated with an increased risk of BC, and this risk is higher among muscle invasive expressing low FGFR3 levels. Herein, we found that serum 25(OHD) deficiency/insufficiency occurred in 75% and 80% in non-muscle and muscle invasive bladder cancer patients, respectively. However, NMIBC patients also showed a significant higher level compared to MIBC patients’ group 17.49±5.04 and 13.17±4.5 ng/mL, respectively (p<0.001). Our findings need to be validated in a larger cohort.

Vitamin D could modulate cancer prognosis. A recent meta-analysis concluded that higher plasma 25(OHD) is associated with better overall survival for breast, haematological and colorectal cancers and better progression-free for breast, haematological and skin cancer [19]. Vitamin D could promote better cancer prognosis by inhibition of tumor growth, induction of differentiation, regulation of immune function, promotion of autophagy, and anti-inflammatory effects [20].

Ben Fradj et al. [21] followed 177 patients with NMIBC over 6 years. They found that recurrence, progression, and cancer-specific mortality risk were higher in patients with the lower plasma 25(OHD). In our trial, there was no significant relationship between serum levels of 25(OHD) and recurrence rate. These opposing results could be attributed to the short-term follow up period and a smaller sample size in our study. A larger cohort with a long-term follow up is needed.

This study has some limitations; serum 25(OHD) concentrations were assessed only once at the time of diagnosis and serum 25(OHD) concentrations could be changed over time during follow up, and thus could affect the outcomes. In addition, none of serum 25(OHD) deficiency/insufficiency patient had received vitamin D supplements or fortified food that could have influenced the recurrence rate. Also, we had only a short-term follow up that cannot precisely assess other prognostic values as progression and cancer-specific mortality.

## Conclusion

Serum 25(OHD) is significantly decreased in bladder cancer patients, especially among those with muscle invasive tumor. However, baseline serum 25(OHD) does not predict recurrence in non-muscle invasive bladder cancer.

## Supporting information

S1 Table Reagents and working solutions used for measurements of 25(OH)D. (DOCX)

S1 Fig Serum 25-hydroxyvitamin D (mean SD) in our patients. (DOCX)
