# Supplementary material for: A prospective trial investigating the role of Serum 25-Hydroxyvitamin D in diagnosis and prognosis of bladder cancer
Source: PLoS One. 2022 Jun 16;17(6):e0266371. doi: 10.1371/journal.pone.0266371 (PMC9202854; doi:10.1371/journal.pone.0266371)
Supplement: S1 Table — (DOCX) [file pone.0266371.s001.docx]

**S1 Table. Reagents and working solutions used for measurements of 25(OH)D.**

| PT1: Pre-treatment reagent 1 (white cap) | 4 mL: Dithiothreitol 1 g/L, pH 5.5 |
| --- | --- |
| PT2: Pre-treatment reagent 2 (gray cap) | 4 mL: Sodium hydroxide 28 g/L |
| M: Streptavidin-coated microparticles (transparent cap) | 6.5 mL: Streptavidin- coated microparticles 0.72mg/Ml ; preservative |
| R1: Vitamin D binding protein‑Ru/(bpy) (gray cap) | 6.5 mL: Ruthenium labelled vitamin D binding protein 100 μg/L; bis‑tris propane buffer 100 mmol/L; albumin (human) 40 g/L; pH 6.4;preservative |
| R2: 25‑hydroxyvitamin D~biotin (black cap) | 6.5 mL: Biotinylated 25‑hydroxyvitamin D 140 μg/L; bis‑tris propane buffer 100 mmol/L; pH 8.6; preservative |
