# Supplementary material for: A prospective trial investigating the role of Serum 25-Hydroxyvitamin D in diagnosis and prognosis of bladder cancer
Source: PLoS One. 2022 Jun 16;17(6):e0266371. doi: 10.1371/journal.pone.0266371 (PMC9202854; doi:10.1371/journal.pone.0266371)
Supplement: S1 Fig — (DOCX) [file pone.0266371.s002.docx]

|  |  |
| --- | --- |

|  | **± 0.87** |
| --- | --- |

**S 1 Fig:** Serum 25-hydroxyvitamin D (mean SD) in our patients.
